# Identification and Characterization of CXCR4-Positive Gastric Cancer Stem Cells

**DOI:** 10.1371/journal.pone.0130808

**Published:** 2015-06-25

**Authors:** Takeshi Fujita, Fumiko Chiwaki, Ryou-u Takahashi, Kazuhiko Aoyagi, Kazuyoshi Yanagihara, Takao Nishimura, Masashi Tamaoki, Masayuki Komatsu, Rie Komatsuzaki, Keisuke Matsusaki, Hitoshi Ichikawa, Hiromi Sakamoto, Yasuhide Yamada, Takeo Fukagawa, Hitoshi Katai, Hiroyuki Konno, Takahiro Ochiya, Teruhiko Yoshida, Hiroki Sasaki

**Affiliations:** 1 Department of Translational Oncology, National Cancer Center Research Institute, Tokyo, Japan; 2 Division of Molecular and Cellular Medicine, National Cancer Center Research Institute, Tokyo, Japan; 3 Department of Clinical Genomics, National Cancer Center Research Institute, Tokyo, Japan; 4 Division of Genetics, National Cancer Center Research Institute, Tokyo, Japan; 5 Gastrointestinal Medical Oncology, National Cancer Center Hospital, Tokyo, Japan; 6 Gastric Surgery Division, National Cancer Center Hospital, Tokyo, Japan; 7 Division of Translational Research, Exploratory Oncology Research & Clinical Trial Center, National Cancer Center, Chiba, Japan; 8 Kanamecho Hospital, Tokyo, Japan; 9 Second Department of Surgery, Hamamatsu University School of Medicine, Shizuoka, Japan; Okayama University, JAPAN

## Abstract

Diffuse-type solid tumors are often composed of a high proportion of rarely proliferating (i.e., dormant) cancer cells, strongly indicating the involvement of cancer stem cells (CSCs) Although diffuse-type gastric cancer (GC) patients have a poor prognosis due to high-frequent development of peritoneal dissemination (PD), it is limited knowledge that the PD-associated CSCs and efficacy of CSC-targeting therapy in diffuse-type GC. In this study, we established highly metastatic GC cell lines by *in vivo* selection designed for the enrichment of PD-associated GC cells. By microarray analysis, we found C-X-C chemokine receptor type 4 (CXCR4) can be a novel marker for highly metastatic CSCs, since CXCR4-positive cells can grow anchorage-independently, initiate tumors in mice, be resistant to cytotoxic drug, and produce differentiated daughter cells. In clinical samples, these CXCR4-positive cells were found from not only late metastasis stage (accumulated ascites) but also earlier stage (peritoneal washings). Moreover, treatment with transforming growth factor-β enhanced the anti-cancer effect of docetaxel via induction of cell differentiation/asymmetric cell division of the CXCR4-positive gastric CSCs even in a dormant state. Therefore, differentiation inducers hold promise for obtaining the maximum therapeutic outcome from currently available anti-cancer drugs through re-cycling of CSCs.

## Introduction

Gastric cancer (GC) is one of the leading causes of cancer-related deaths worldwide. Histopathologically, GCs can be classified into two major categories: intestinal-type and diffuse-type. Intestinal-type GC predominates in high-risk geographic areas and develops through some sequential stages including *Helicobacter pylori* (*H*. *pylori*)-associated atrophic gastritis, intestinal metaplasia (IM), and dysplasia. By contrast, diffuse-type GC, which accounts for half of all GC patients, has a wide geographical distribution and develops even from *H*. *pylori*-free, morphologically normal gastric mucosa without atrophic gastritis or IM, and diffuse-type GC may differ from the intestinal-type both genetically and phenotypically [1–5]. Unlike the decreasing incidence of the intestinal-type, the prevalence of the diffuse-type is reportedly increasing worldwide [6]. The prognosis for advanced diffuse-type GC patients has remained poor over the past decade because of a high rate of peritoneal dissemination (PD) (78%) compared to that of intestinal-type (45%) [7]. In particular, patients with Bormann type IV GC have an extremely poor prognosis, even if they received multidisciplinary treatment [8]. Therefore, addressing PD is an urgent issue for improvement in the prognosis of diffuse-type GC patients.

Diffuse-type GCs are typically characterized by an extensive stromal fibrosis with poor vascularity and rare proliferative (*i*.*e*. dormant) cancer cells, which leads to a substantial therapeutic resistance. From the viewpoint of chemotherapy, transforming growth factor β (TGF-β) has been thought to contribute to the aggressive progression via epithelial-mesenchymal transition and by inducing fibrosis [9]. However, TGF-β also inhibits cell proliferation, induces apoptosis, and mediates differentiation in epithelial cells, suggesting that components of the TGF-β signaling pathways have tumor-suppressive activity in epithelial tumors [10, 11]. Although accumulating evidence has demonstrated the critical role of TGF-β in cancer stem cell (CSC) biology, there is limited information regarding its role in diffuse-type GCs.

It has been reported that each population of tumor cells show functional heterogeneity characterized by distinct proliferation and differentiation capacity in diverse types of tumors [12]. To account for such tumor heterogeneity, two models have been proposed: the clonal evolution model [13] and the CSC model [14]. The latter model has received wide attention, since it provides a reasonable explanation for resistance to conventional chemo- and radiotherapy, in which quiescent or slow-cycling CSCs survive, and results in eventual tumor relapse [15–17]. Therefore, therapeutic strategies to target CSCs are imperative to eradicate residual disease and to prevent recurrence in not only GC but also other cancers. Furthermore, with current insights into the cellular mechanisms of carcinogenesis, the metastatic potential of tumor cells is attributed to CSCs, which can clonally initiate tumor formation at distant sites [18 19]. Although a number of cell surface molecules have been proposed as CSC markers in a large variety of human malignancies, there has been no marker identification for PD-associated CSCs in GCs [20–23]. To identify such markers, we considered the notion that functionally and genetically heterogeneous tumors have common and intrinsic mechanisms of tumor maintenance according to the context of a given microenvironment. Accordingly, we hypothesized that CSCs should be defined functionally by well-validated assays such as *in vivo* transplantation. In diffuse-type GC, we initially focused on the peritoneum-specific colonization of cells and enriched the PD-associated CSCs by repetitive *in vivo* selections [24, 25]. Here, we found C-X-C chemokine receptor type 4 (CXCR4) can be a marker for PD-associated gastric CSCs and demonstrated that TGF-β enhances the efficacy of anti-tumor drugs via induction of cell differentiation /asymmetric cell division in the CXCR4^+^ CSC population even in a dormant state.

## Materials and Methods

### Clinical samples

Clinical samples were provided by the National Cancer Center Hospital (Tokyo, Japan) after obtaining written informed consent from each patient and approval by National Cancer Center Institutional Review Board (ID: 15–44, 2012–181, 2010–031).

### Cell lines and primary cultures

A human GC cell line, HSC-60 was established by a collaborator using the procedure as described previously [26]. A highly peritoneal-metastatic cell line, 60As6 was established from HSC-60 using orthotopic tissue implantation into SCID/SCID mice as briefly follows: the xenografted tumor of HSC-60 cells was transplanted into the gastric wall of a mouse. We repeated six cycles of harvesting ascitic tumor cells and the orthotopic inoculation of these cells. These two cell lines were maintained in an RPMI-1640 medium supplemented with 10% fetal calf serum. We also established two luciferase-expressing cell lines (HSC-60Luc and 60As6Luc). Six other GC-derived cell lines (HSC-39, HSC-44, 44As3, HSC-58, 58As9, and KATO III) were also maintained under the same condition. Of these six, HSC-39, HSC-44, 44As3, HSC-58, and 58As9 were established by the above procedure [27, 28], and KATO III was obtained from American Type Culture Collection. For primary cultures, cells were collected from patients’ peritoneal washings and ascites (NSC-16C, NSC-20C, and NSC-22C) and cultured in an RPMI-1640 medium supplemented with 10% fetal calf serum.

### Microarray analysis

Total RNA of 60As6, 60As6Luc, HSC-60, and HSC-60Luc cells was isolated by suspending the cells in an ISOGEN lysis buffer (Nippon Gene, Toyama, Japan) followed by isopropanol precipitation. We conducted microarray analyses twice by using Human Genome U133 Plus 2.0 Array (Affymetrix, Santa Clara, CA). The procedures were conducted according to the suppliers’ protocols. The arrays were scanned with a GeneChip Scanner 3000 (Affymetrix), and the data were analyzed by Microarray Suite version 5.0 with Affymetrix default analysis settings and global scaling as normalization method. The trimmed mean target intensity of each array was arbitrarily set to 1000. All the microarray data have been deposited in a MIAME compliant database, GEO; accession number GSE53276. By a 2-fold change, 684 genes were selected as specific genes for 60As6 and 60As6Luc cells, and 1150 genes were selected for HSC-60 and HSC-60Luc cells.

### Animal experiments

Six-week-old female C.B17/Icr-scid (SCID/SCID) mice (CLEA Japan, Japan) were bred at a room temperature with a 12 h light/dark daily cycle. The mice were maintained under specific pathogen-free conditions and provided sterile food, water, and cages. 1×10^4^ to 1 × 10^6^ of cancer cells were suspended with 1 ml of phosphate-buffered saline (PBS) and then injected into the abdominal cavity by use of a 26 1/2-gauge needle. Mice were weighed and measured weekly. Humane endpoint criteria included significant accumulation of abdominal ascites, dyspnea, piloerection, anemia, or weight loss exceeding 10% of initial weight. All experiments were conducted in accordance with the ethical guidelines of the International Association for the Study of Pain and were approved by the Committee for Ethics in Animal Experimentation of the National Cancer Center. Efforts were made to minimize the numbers and any suffering of animals used in the experiments.

### RT-PCR

Total RNA was isolated by suspending the cells in an ISOGEN lysis buffer followed by isopropanol precipitation. Semi-quantitative RT-PCR was carried out within linear range from 25 to 30 cycles for *MUC5AC*, *CXCR4*, *CXCR7*, *CXCL12*, *LGR5*, *TGFBR1*, *TGFBR2*, and *ACTB* using following primers: 5’-ACATGTGTACCTGCCTCTCT-3’ and 5’-GTTGTCCACATGGCTGTT-3’ for *MUC5AC*, 5’-CCACTGAGTCTGAGTCTTCA-3’ and 5’-AGACTGTACACTGTAGGTGC-3’ for *CXCR4*, 5’-CGGAGTACTCTGCCTTGGAG-3’ and 5’-ACAGCTGCGTCATCAAGAGA-3’ for *CXCR7*, 5’-GAAGCTTCCCTGACTCATTCT-3’ and 5’-AAAGCACGCTGCGTATAGGA-3’ for *CXCL12*, 5’-TGCTCTTCACCAACTGCATC-3’ and 5’-CTCAGGCTCACCAGATCCTC-3’ for *LGR5*, 5’-GCATGATGGACCTGTCTACA-3’ and 5’-GCTTCATATCCTGGTGATGTC-3’ for *TGFBR1*, 5’-CAAAGGTCCCATTTGCAGTT-3’ and 5’-GAGACCTTCCACCATCCAAA-3’ for *TGFBR2*, and 5’-GAAGTCCCTTGCCATCCTAA-3’ and 5’-GCACGAAGGCTCATCATTCA-3’ for *ACTB*.

### Immunocytochemistry

Indirect fluorescent immunocytochemistry was performed for CXCR4, MUC5AC, and Smad2/3 on cells grown in 4-well chamber slides. Cells were fixed in 4% paraformaldehyde at room temperature for 20 min. After three times washing in PBS(-), they were incubated with anti-human CXCR4 monoclonal antibody (R&D Systems, Minneapolis, MN), anti-human MUC5AC antibody (Santa Cruz Biochemistry, Santa Cruz, CA), or anti-human Smad2/3 antibody (BD Biosciences, Bedford, MA) followed by incubation with a 1:1000 dilution of donkey anti-mouse Alexa 488-conjugated serum (Invitrogen, Grand Island, NY) for 1 hr at room temperature. Cell nuclei were stained with 4’, 6-diamidino-2-phenylindole dihydrochloride (DAPI) (Invitrogen, Carlsbad, CA). For cell-lineage assay, sorted single CXCR4^+^ small cells were stained by CellTracker Green (Invitrogen) for tracing the viable cells.

### Flow cytometry and cell sorting

60As6 cells (1×10^6^ cells) were co-incubated for 30 min at 4°C with the following antibodies: APC anti-human CD184 (CXCR4) (IgG2a, clone 12G5) or APC Mouse IgG2a, k Isotype control obtained from BioLegend (San Diego, CA). Dead cells were labeled with propidium iodide and excluded from the analysis. In cell sorting, the CXCR4-positive and CXCR4-negative subpopulations were purified using a JSAN cell sorter (Bay Bioscience, Kobe, Japan), and were analyzed by a software, FlowJo (Tree Star, Inc., Ashland, OR). For cell cycle analyses, cells were transferred to a serum-free medium for 5 days, and subsequently harvested with 0.05% trypsin-EDTA, and then suspended in cold 80% ethanol. After fixing overnight at -20°C, the samples were washed in PBS(-) and incubated in 500μl of PI/RNase Staining Buffer (BD Pharmingen, San Diego, CA) per 1×10^6^ cells for 30 min at room temperature before analysis. Flow cytometry was performed using FACSCalibur (BD, Franklin Lakes, NJ), and analyzed by a software, FlowJo.

### Anchorage-independent cell growth assay

A soft agar colony formation assay was carried out using the CytoSelect 96-Well Cell Transformation Assay Kit (Cell Biolabs, San Diego, CA) following the instructions of the manufacturer. Briefly, 5×10^3^ CXCR4-positive or CXCR4-negative small cells were suspended in DMEM containing 0.4% low-melting agarose and 10% FBS and seeded onto 1% of low-melting agarose containing DMEM and 10% FBS. After incubating the cells for 5 days at 37°C in 5% CO_2_, cells were lysed and detected with CyQuant GR dye. The fluorescence of the colony forming cells was read in Multifunctional Microplate Reader (Safire, Tecan, Mannedorf, Swizerland) at excitation/emission wave lengths of 485/520 nm.

### Invasion assay

Cell invasion was determined using BD BioCoat Tumor Invasion Assay System (BD Biosciences) according to the instructions of the manufacturer. Briefly, 4×10^5^ of 60As6 cells with serum-free media were seeded into the upper chamber of the system. Bottom wells were filled with media with 10 ng/ml SDF-1β (Invitrogen, Carlsbad, CA). After 24 hr incubation, the cells in the upper chamber were removed and the cells that invaded through the Matrigel membrane were stained with Diff-Quick stain (BD Biosciences) and counted manually.

### Caspase assay

The 60As6 cells were prepared in a 96-well plate (5×10^3^ cells/well). The caspase 3/7 assay was performed using the Caspase-Glo 3/7 Assay (Promega, Madison, WI) at 24 hr after the addition of recombinant TGF-β1 (240-B, R&D systems) into the cell culture at a concentration of 2 ng/ml.

### Cell growth assays

To determine IC_50_ values against docetaxel (Doc), both HSC-60 and 60As6 cells were cultured in the presence of 0–100 nM Doc (Aventis Pharma, Tokyo, Japan) for 4 days at 37°C in 5% CO_2_ followed by the conventional MTT assay. For validating the combination treatment with Doc and TGF-β, all of 60As6 cells and 2 primary cultures (NSC-16C and -22C) was prepared in a 6-well plate (1×10^5^ cells/well) followed by a treatment with Doc (2.5 nM) and TGF-β1 (0 or 2 ng/ml) for 72 hr at 37°C in 5% CO_2_. Cells were stained by trypan blue and counted with TC20 Automated Cell Counter (Bio-Rad, Hercules, CA).

### Statistical analysis

All data were expressed as the mean ± SE, and analyzed using the unpaired t-test. The accepted level of significance was *p*<0.05. SPSS (SPSS, Chicago, IL) was used for all statistical analyses.

## Results

### Phenotypic differences between parental HSC-60 cells and the highly tumorigenic subline 60As6 cells

To address the phenomenon of diffuse-type GC-associated PD from the viewpoint of molecular and cellular biology, we established a highly peritoneal-metastatic cell line, 60As6, from a diffuse-type GC derived cell line, HSC-60, through *in vivo* selections consisting of an orthotopic inoculation of the tumor cells and the isolation of highly-metastatic ones from the ascites of immunodeficient mice [25]. Although the doubling time of these two cell lines was comparable (30 hr for HSC-60 and 31 hr for 60As6) *in vitro*, 60As6 cells formed multiple tumor nodules more quickly after intraperitoneal inoculation into the mice. The median survival time was 167 days after implantation of 5×10^6^ HSC-60 cells, whereas the implantation of the same number of 60As6 cells resulted in a remarkable formation of bloody ascites and the median survival time was only 28 days. Anchorage-independency may be required for the survival of free cancer cells in the peritoneal cavity and for colonization. In colony formation assay, 60As6 cells formed many more colonies compared to HSC-60 cells (S1 Fig) showing that 60As6 possesses a high ability of anchorage-independent growth. We validated the chemoresistance also known as a major property of CSCs. The cells were cultured in the presence of docetaxel (Doc), which is a frequently employed anti-cancer drug for the treatment of GC patients. 60As6 showed a 6-fold higher IC_50_ value of Doc compared to that of HSC-60 (52 nM and 8.4 nM, respectively) (S2 Fig). These data indicate that 60As6 has gained a high chemoresistant phenotype. Furthermore, the morphology of HSC-60 cells was characterized as flat and large, whereas that of 60As6 cells was semi-floating and small, which is similar to the morphology often observed for CSCs (S3 Fig). We next examined the lipid rafts localization using Cholera toxin B subunit (CTxB) as a lipid raft marker, since it has already been reported that lipid raft clusters were enriched in hematopoietic stem cells [29]. An accumulation of the fluorescent CTxB was observed for 60As6 cells but not in HSC-60, suggesting another CSC-like property for the former cells (S3 Fig).

To assess the biological differences between HSC-60 and 60As6 cells, we compared gene expression profiles by microarray analysis (S1 Table). Down-regulation of three gastric pit cell-differentiation markers (*MUC5AC*, *MUC1*, and *TFF1*) and some cytokeratins were found in 60As6. By contrast, several stem cell markers (*LY75*, *CD44*, *GPNMB*, and *CXCR4*) were upregulated in this cell line. The expression profile suggests that 60As6 has a strong mesenchymal phenotype, which is a characteristic of epithelial cell-derived CSCs.

**Fig 1:**
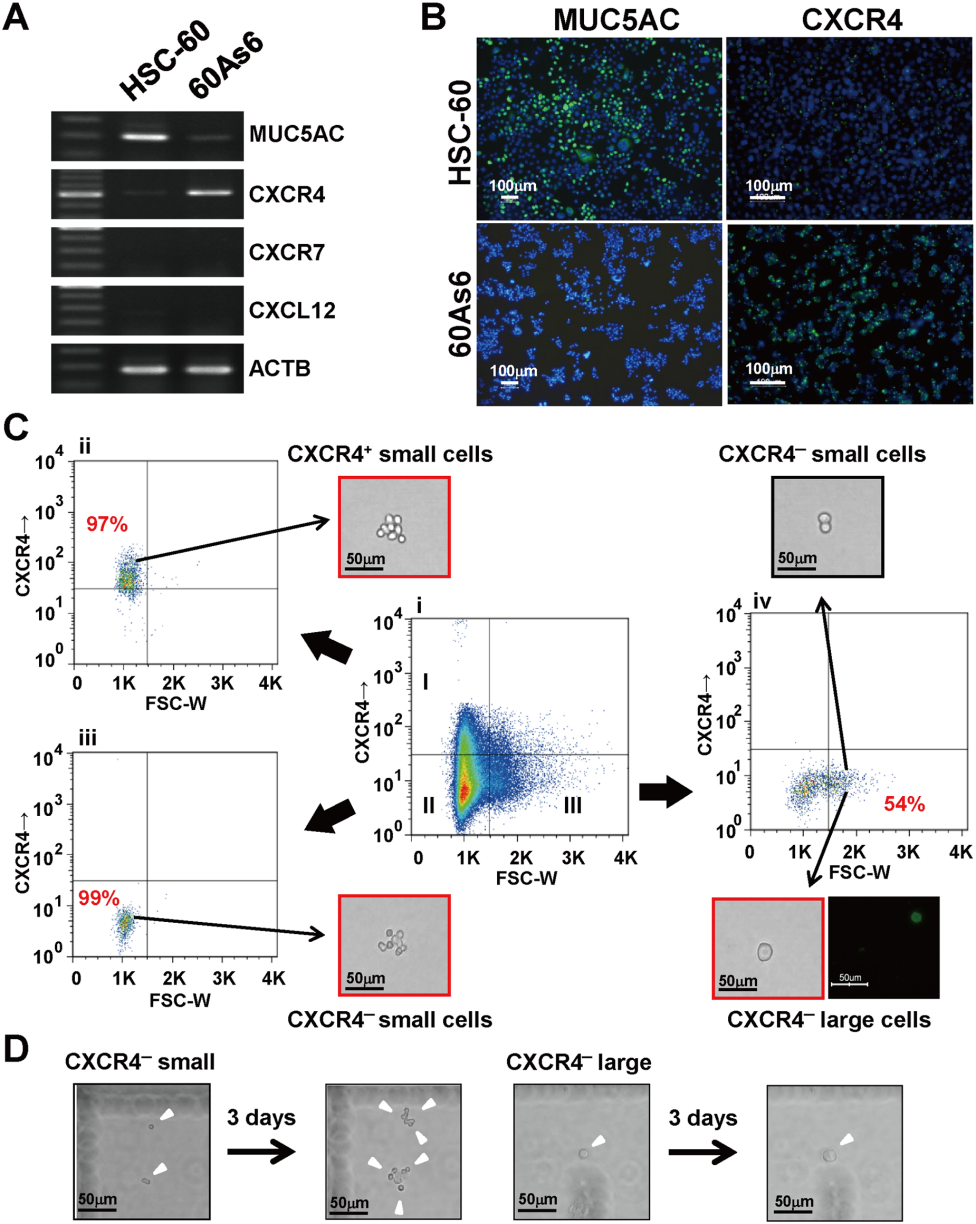
Expression of *CXCR4* is upregulated after serial transplantation. (A) RT-PCR of *MUC5AC*, *CXCR4*, *CXCR7*, and *CXCL12* in a GC cell line HSC-60 and its subline 60As6. (B) Immunocytochemistry for MUC5AC (left panel, green) and CXCR4 (right panel, green) in HSC-60 and 60As6 cells, respectively. Nuclei were counterstained with DAPI (blue). Scale bars represent 100μm. (C) Flow cytometry analysis of cell-surface CXCR4 in 60As6 cells. The inner panels represent CXCR4^+^ small cells (I), CXCR4^-^ small cells (II) and CXCR4^-^ large cells (III) (Ci). Each cell population was separated by FACS at its respective purity (Ci-Civ). Immunopositive cells for MUC5AC were observed in only the CXCR4^-^ large cell fraction (Civ, lower panel, green). Scale bars represent 50μm. (D) Representative images after culturing CXCR4^-^ small and large cells for 3 days. Scale bars represent 50μm.

We further investigated the mRNA expression of both a typical differentiation marker *MUC5AC* [3, 4] and a metastasis-associated chemokine receptor *CXCR4* [30] by RT-PCR. In accordance with the microarray results, *MUC5AC* and *CXCR4* mRNAs showed a mutually exclusive expression in HSC-60 and 60As6 (Fig 1A). A chemokine SDF-1 encoded by *CXCL12* is known to bind CXCR4 and its cognate receptor CXCR7 [30]; however, the mRNA of *CXCL12* and *CXCR7* were not detected in either cell line. The expression of both MUC5AC and CXCR4 was also confirmed at a protein level by immunocytochemical staining (Fig 1B). Thus, these results suggest that the HSC-60 cells harbor more differentiated cells than do the 60As6 cells, which are mostly composed of undifferentiated cells. Taken together, all these CSC-like characteristics of 60As6 indicate that the undifferentiated subpopulation with a high tumorigenicity and drug-resistance could be efficiently enriched from parental cells during *in vivo* selections.

### Identification and characterization of gastric CSCs in 60As6 and primary cultured cells

We next examined the role of CXCR4 in diffuse-type GC cells for PD. As shown in Fig 1A, the receptor *CXCR4* was highly expressed in 60As6 compared with HSC-60, whereas their ligand *CXCL12/SDF1* was not in both. However, it is noteworthy that this ligand is known to be expressed in peritoneal mesothelial cells [31] and to function as a chemokine to induce distant metastasis through an interaction with CXCR4-expressing cancer cells [30]. Therefore, we examined whether CXCR4 might serve as a marker for identifying PD-associated CSCs. The expression of CXCR4 on 60As6 cells was analyzed by fluorescence activated cell sorting (FACS) analysis. According to the CXCR4 expression and forward scattering pattern, 60As6 cells exhibited a heterogeneity consisting of three different subpopulations: I. CXCR4^+^ small cells (15%), II. CXCR4^-^ small cells (65%), and III. CXCR4^-^ large cells (20%) (Fig 1Ci). The cells were then separated to each subpopulation with a purity of I: 97%, II: 99%, and III: 54%, respectively (Fig 1Cii–1Civ). The low sorting purity of the CXCR4^-^ large cell subpopulation III was attributable to the contamination with aggregated CXCR4^-^ small cells (Fig 1Civ, black-boxed panel). Among these three subpopulations, only a CXCR4^-^ large cell subpopulation contained MUC5AC^+^ differentiated cells (Fig 1Civ, three outer panels). By cell growth monitoring in a single cell level, it was found that CXCR4^-^ small cells proliferated, whereas the CXCR4^-^ large cells did not undergo cell division even 3 days after culture (Fig 1D and S4 Fig). These findings suggest that CXCR4^-^ small cells are transient amplifying cells, while the CXCR4^-^ large cells are terminally-differentiated cells.

**Fig 2:**
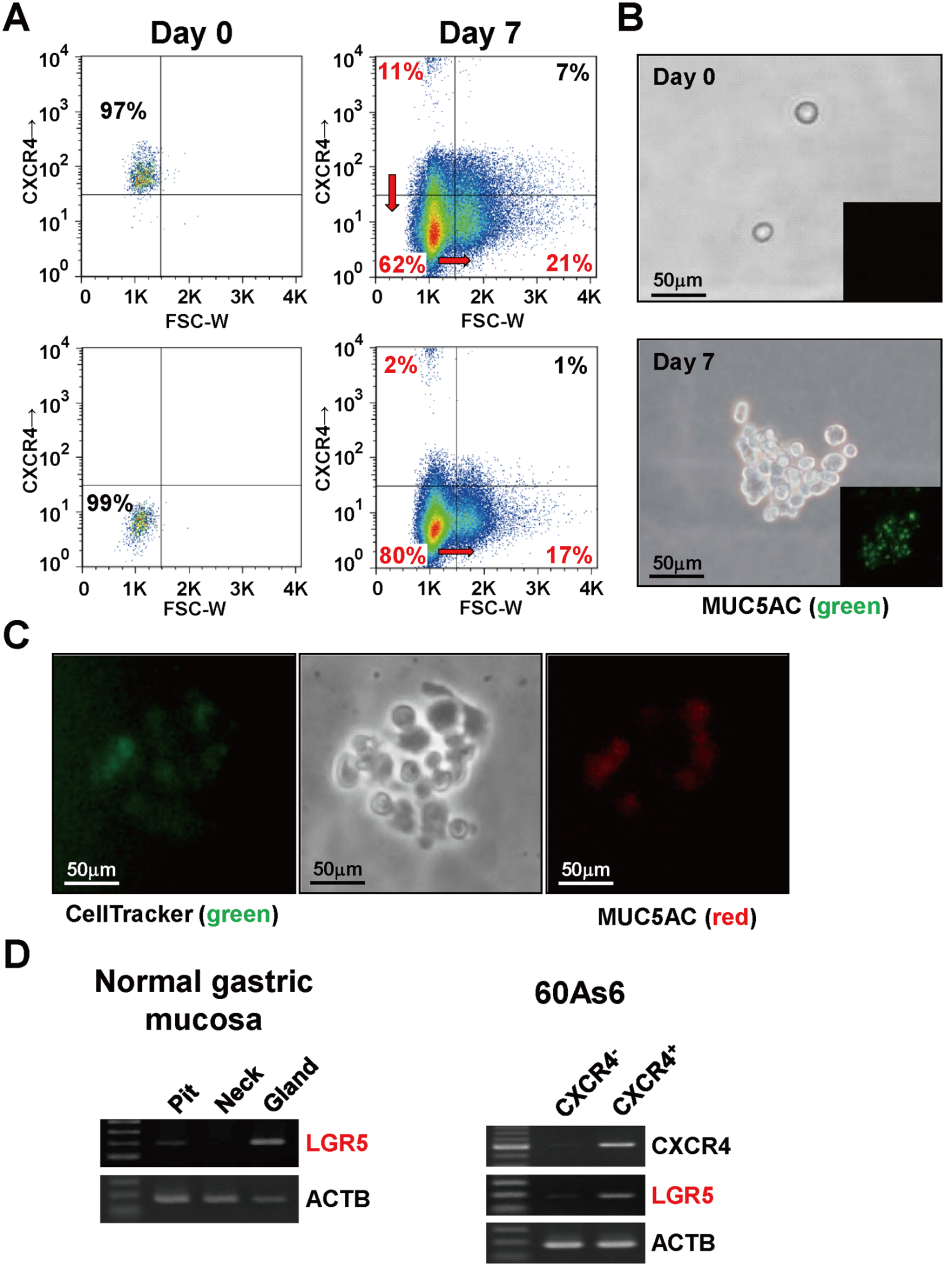
CXCR4^+^ small cells are required for repopulation of all subsets of 60As6 cells. (A) Both CXCR4^+^ and CXCR4^-^ 60As6 cells were sorted by FACS (left column). The cells of each fraction were cultured for 7 days and then analyzed for the expression levels of CXCR4 (right column). (B) Immunocytochemistry for MUC5AC (green) of a colony derived from a single CXCR4^+^ small cell. Scale bars represent 50μm. (C) Cell-lineage assay with two different colors. Sorted single CXCR4^+^ small cells were stained by anti-MUC5AC antibody (red) and CellTracker Green (Invitrogen, green) and for tracing the viable cells for 7 days. Scale bars represent 50μm. (D) RT-PCR of *LGR5* in normal gastric epithelial cells (left panel), CXCR4^+^ small cells and CXCR4^-^ small cells (right panel). Laser microdissection separates the normal gastric mucosa into three regions: pit, neck, and gland.

After sorting the two subpopulation (I and II), we analyze any shift of the cell population (Fig 2A); after 7 days of culture. CXCR4^+^ small cells corresponding to subpopulation I generated themselves, CXCR4^-^ small cells, and CXCR4^-^ large cells (11%, 62%, and 21%, respectively), whereas CXCR4^-^ small cells corresponding to subpopulation II were able to give rise mainly to themselves (80%) with some large cells (17%) and very few CXCR4^+^ small cells (2%). These results suggest that CXCR4^+^ small cells have differentiation potential and produce CXCR4^-^ large cells possibly through a stage of CXCR4^-^ small cells in the scheme of cell differentiation.

Furthermore, a CXCR4^+^ small single cell produced a colony containing MUC5AC^+^ differentiated cells after 7 days in culture (Fig 2B). This finding was supported by a cell-lineage assay combined with live cell staining and immunostaining of MUC5AC (Fig 2C). In addition, CXCR4^+^ small cells were shown to express a high level of *LGR5*, which is known as a marker of gastric epithelial stem cells located at the gland of normal mucosa [32] (Fig 2D).

**Fig 3:**
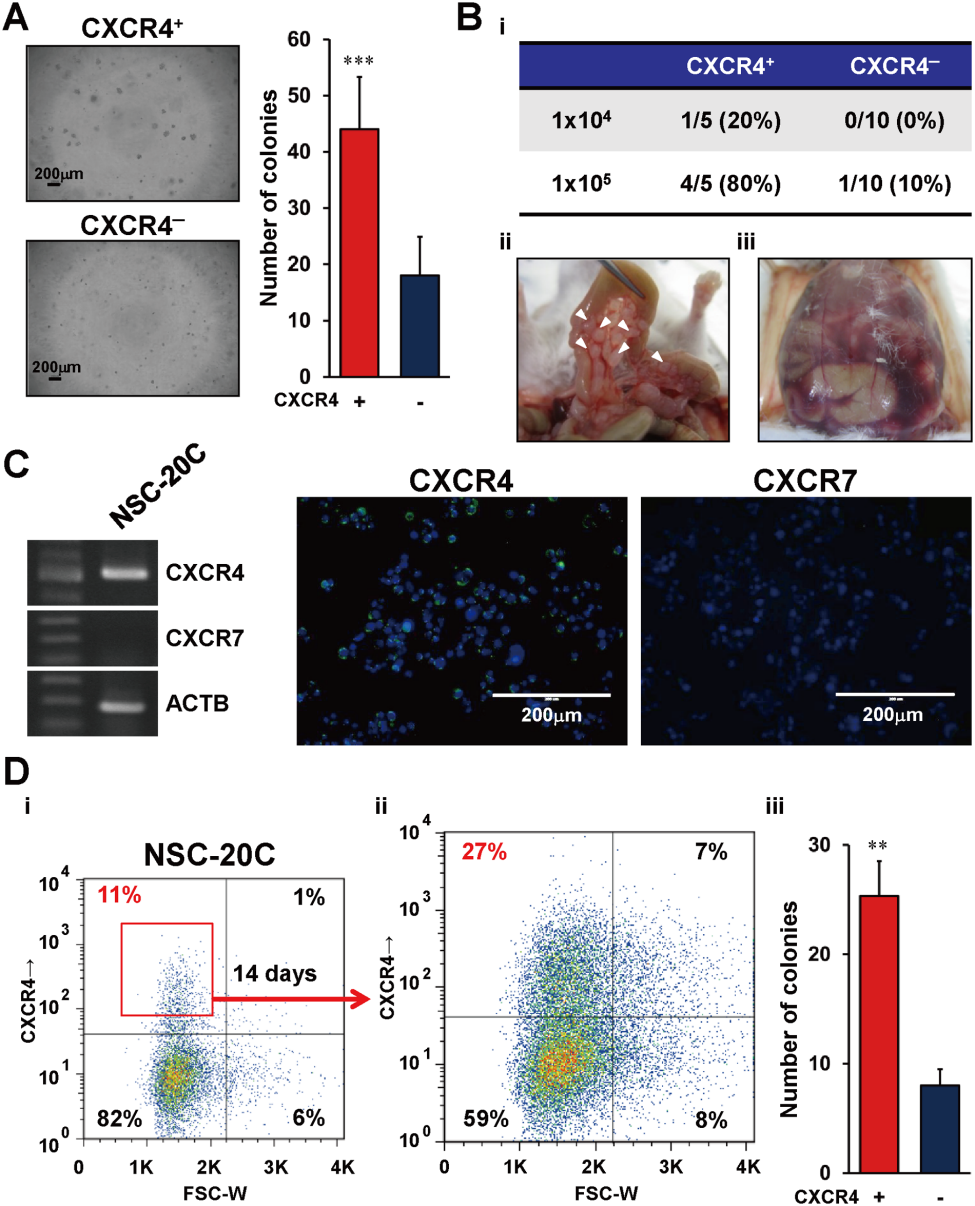
Tumorigenicity of CXCR4^+^ small cells sorted from 60As6 and primary cultured GC cells. (A) Representative images of anchorage independent growth of CXCR4^+^ small cells and CXCR4^-^ small cells in colony formation assay (left). Quantitative analysis of cell growth using CyQuant GR dye (right) (n = 3, mean + SE; ***p<0.001). Scale bars represent 200μm. (B) Tumor formation frequency of CXCR4^+^ small cells and CXCR4^-^ large cells in xenograft mice (Bi). The lower panels show representative macroscopic findings of multiple tumor nodules (Bii, arrowhead) along with the intestine and bloody ascites (Biii) in a SCID/SCID mouse. (C) RT-PCR (left panel) and immunocytochemical staining (right panels) of CXCR4 (green) and CXCR7 (green) in primary cultured GC cells (NSC-20C). Scale bar represents 200μm. (D) Flow cytometric analysis in primary cultured GC cells (NSC-20C) (Di) and sorted CXCR4^+^ cells after cultivating for 14 days (Dii). Colony formation assay of CXCR4^+^ and CXCR4^-^ small cells derived from NSC-20C cells (n = 3, mean + SE; **p<0.005) (Diii).

Next, we validated the anchorage independency and tumor-initiating ability of the two small cell subpopulation (I and II). In the colony formation assay, CXCR4^+^ small cells formed many more colonies than did the CXCR4^-^ small cells (Fig 3A). Serial dilution experiments of intraperitoneal inoculation in immunodeficient mice revealed that CXCR4^+^ small cells also possessed a higher tumorigenic ability (tumor development in 4/5 and 1/5 mice upon inoculation of 10^5^ and 10^4^ cells, respectively) compared with CXCR4^-^ small cells (1/10 and 0/10 mice following 10^5^ and 10^4^ cells, respectively) (Fig 3Bi). Notably, all five tumor-bearing mice after inoculation of CXCR4^+^ small cells had multiple tumor nodules (Fig 3Bii), and began to develop bloody ascites within 3 weeks (Fig 3Biii).

In addition to diffuse-type GC cell lines, we also investigated the role of CXCR4 in a clinical sample (NSC-20C): a primary culture established from the ascites of a diffuse-type GC patient with PD. In RT-PCR and immunostaining experiments, it was confirmed that the primary culture express *CXCR4* but not *CXCR7* (Fig 3C). Importantly, CXCR4^+^ small cells sorted from NSC-20C possessed both a repopulating ability (Fig 3Di and 3Dii) and a high colony formation ability (Fig 3Diii).

### Involvement of the CXCR4/CXCR7/SDF-1 axis in PD

**Fig 4:**
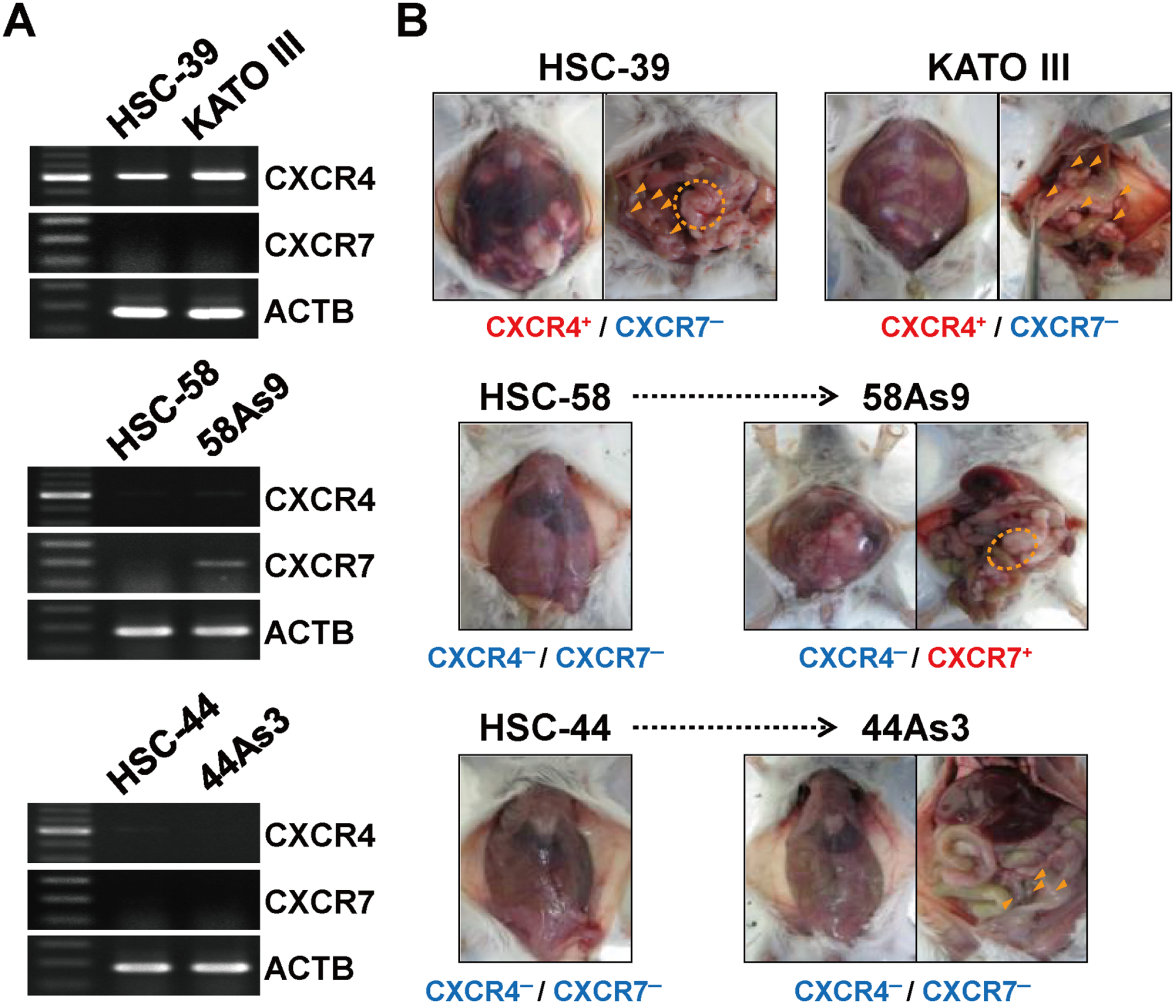
Relationship between tumor phenotypes and *CXCR4* or *CXCR7* expression in GC cell lines. (A) RT-PCR of *CXCR4* and *CXCR7* in 6 diffuse-type GC derived cell lines. (B) Macroscopic findings of bloody ascites and multiple tumor nodules (arrowhead or dotted circle) in SCID/SCID mice formed within 3 weeks after inoculation of each cell line.

In six other diffuse-type GC derived cell lines (HSC-39, HSC-44, 44As3, HSC-58, 58As9, and KATO III), we further investigated the relationship between the expression status of two CXCL12/SDF-1 receptors (CXCR4 and CXCR7) and the accumulation of ascites in mice inoculated with the cancer cells intraperitoneally. The RT-PCR experiments revealed that two GC cell lines (HSC-39 and KATO III) highly expressed *CXCR4* (Fig 4A). Moreover, both of these xenografted mice had multiple tumor nodules and accumulated ascites (Fig 4B). In contrast, the other two cell lines (HSC-44 and HSC-58) showed no or quite low CXCR4 expression, a single or few peritoneal tumor nodule and did not accumulate ascites (Fig 4B). We also confirmed the accumulation of ascites in another highly peritoneal-metastatic subline 58As9 (derived from HSC-58) which expressed *CXCR7* at a high level but not *CXCR4*. One exceptional case was 44As3 derived from HSC44 that had multiple tumor nodules and accumulated ascites without the expression of both *CXCR4* and *CXCR7*. Overall, we found a high correlation between the expression level of *CXCR4* or *CXCR7* and the aggressive phenotypes in seven of the eight cell lines except 44As3.

In clinical practice, peritoneal lavage cytology (CY) provides important prognostic information for GC patients, because it may detect the “seed” of PD before its macroscopic manifestation. Even in the cases with no PD (P0), CY-positive patients have a poor prognosis [33], suggesting that quite a few metastatic CSCs are present in the peritoneal lavage of these patients. Therefore, we next investigated the presence of CXCR4^+^ GC cells in peritoneal washings from the patients without clinically apparent ascites. *CXCR4* mRNA was rarely detected in the washings in 9 of 10 CY-negative patients; however, the mRNA was detected in the washings in 19 of 34 CY-positive patients (S5A Fig). We also confirmed the presence of the CXCR4 and cytokeratin double-positive small GC cells on the cobblestone-shaped mesothelial cells attached to the culture dish surface in a short-term (7 days) culture of the patient’s ascites (S5B Fig). In our experiments, the peritoneal mesothelial cells usually disappeared in long-term culture after about one month. In such long-term culture, we also found the presence of some CXCR4- or CXCR7-highly positive GC cells that showed a characteristics for epithelial-mesenchymal transition, vimentin (VIM)-positive (arrow head in S5C Fig) but cytokeratin (PAN)-negative. Therefore, CXCR4^+^ GC cells are present in the malignant ascites not only in the early stage of peritoneal dissemination (P0/CY^+^) but also in more progressive stages characterized by an overt ascites accumulation. In sum, the above experimental and clinical evidence suggests that a part of CXCR4^+^ or CXCR7^+^ GC cells have a potential for developing peritoneal metastasis.

**Fig 5:**
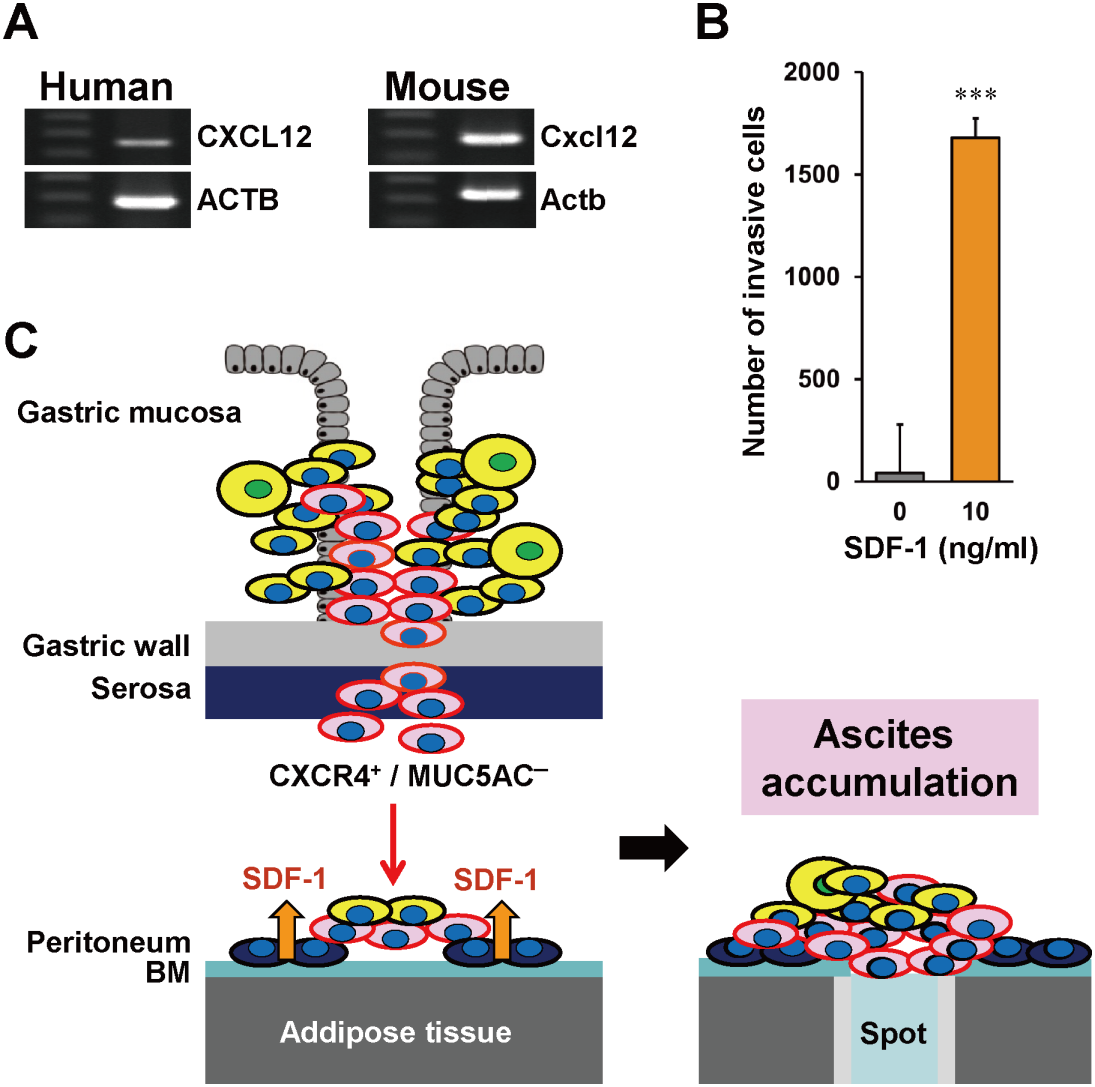
Role of SDF-1 in PD of diffuse-type GC. (A) RT-PCR of *CXCL12* in the peritoneal mesothelium of a human and a SCID/SCID mouse. (B) Cell invasion assay of 60As6 cells in response to SDF-1 (n = 3, mean + SE; ***p<0.001). (C) Schematic diagram for CXCR4/SDF-1-mediated invasion and colonization of isolated cancer cells to the peritoneum. During tumor progression in the primary lesion, cancer cells penetrate the gastric wall and shed from the serosa to the peritoneal cavity. Among them, CXCR4^+^ small cells can survive in the harsh environment and migrate to the peritoneal mesothelium, which expresses SDF-1. Established peritoneal tumor nodes impaired the pump-function of the omental milky spot, which led to the accumulation of malignant bloody ascites.

We next investigated the functional role of the CXCR4 signaling pathway in PD. The expression of *CXCL12*/*SDF-1* in the human and mouse peritoneal mesothelium was confirmed by RT-PCR (Fig 5A). Therefore, the involvement of CXCL12/SDF-1 for cell invasion was investigated. As shown in Fig 5B, the number of invasive 60As6 cells was drastically increased by SDF-1, indicating that these cells have the chemotaxis ability to SDF-1. Lentiviral shRNA-induced knockdown of CXCR4 in 60As6 attenuated SDF-1-mediated invasion but not affected the tumor nodule formation in the peritoneal cavity of mice (not shown). Moreover, forced CXCR4 expression in a parental cell line HSC-60 never increased the tumorigenicity (not shown). Taken together, our data suggest that the CXCR4 is involved in the attachment to the peritoneum but not in the tumor nodule formation.

We showed schematically processes of direct PD from gastric wall in advanced GC patients through the interaction between CXCR4^+^ or CXCR7^+^ CSCs and the SDF-1-expressing peritoneum (Fig 5C). At the first step of metastasis, the CXCR4^+^ CSCs were shed out of the gastric wall into the peritoneal cavity. Subsequently, these cells attach to the basement membrane (BM) of the peritoneum in response to the mesothelium-derived chemoattractant SDF-1 and colonize to form nodules. These events occur at numerous sites over the peritoneal surface, resulting in a blockade of pump-function and then in the accumulation of the ascites.

### TGF-β promotes cell differentiation/asymmetrical cell division of CXCR4^+^ gastric CSCs

**Fig 6:**
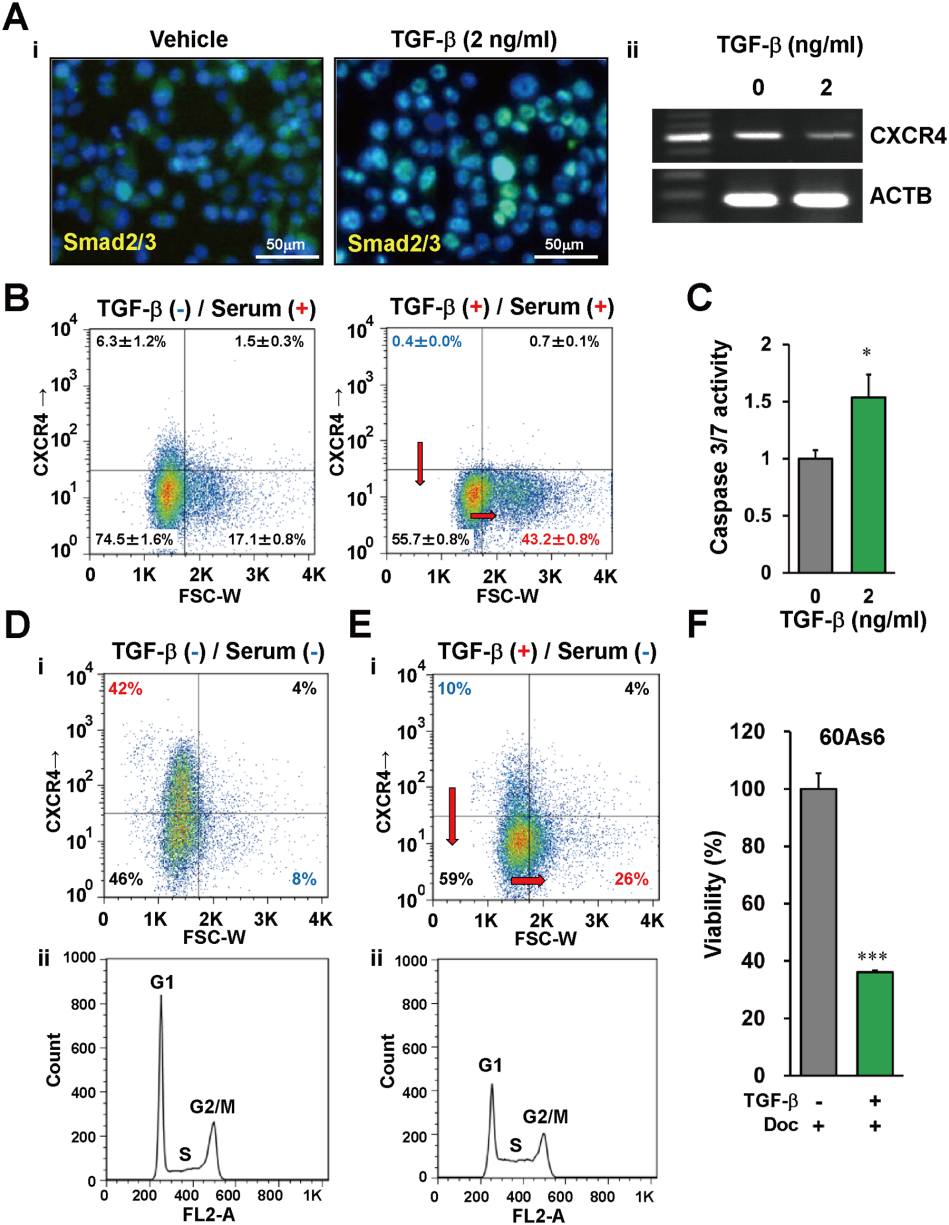
TGF-β promotes differentiation of CXCR4^+^ small cells and enhances the effect of anti-tumor drugs. (A) Smad2/3 nuclear localization (Ai, green) and *CXCR4* expression (Aii) in TGF-β-treated 60As6 cells analyzed by immunohistochemistry and RT-PCR. Nuclei were counterstained with DAPI (blue). Scale bars represent 50μm. (B) Flow cytometry analysis of 60As6 cells with TGF-β treatment under a normal nutrient condition (10% FBS). (C) Caspase 3/7 assay in 60As6 cells with or without TGF-β treatment (n = 3, mean + SE; *p<0.01). (D) Flow cytometry analyses of 60As6 cells with TGF-β treatment under a serum starvation (0% FBS) for 5 days. Dot plotting of the cells (Di). Propidium iodide-based cell cycle analysis (Dii). (*e*) Flow cytometry analyses of 60As6 cells with TGF-β treatment under a serum starvation (0% FBS) for 3 days. Dot plotting of the cells (Ei). Propidium iodide-based cell cycle analysis (Eii). (F) The viability of 60As6 cells after Doc treatment with or without TGF-β (n = 3, mean + SE; ***p<0.001).

TGF-β has been reported to diminish the side population of some diffuse-type GC cell lines [34]. Therefore, we investigated whether TGF-β has a potential to change the characteristics of CXCR4^+^ diffuse-type GC cells. We first examined the expression levels of TGF-β receptors in 60As6 cells and GC primary cultures derived from two independent patients (NSC-16C and -22C). RT-PCR experiments showed that both of two TGF-β receptor genes (*TGFBR1* and *TGFBR2*) expressed in all of these cells (S6 Fig). We also examined the localization of SMAD2/3, since these transcriptional factors are widely known to accumulate in the nuclei in response to the activation of TGF-β signaling. As shown in Fig 6Ai, the nuclear accumulation of SMAD2/3 was detected in 60As6 cells after a 30 min treatment with 2 ng/ml TGF-β.

We next investigated how CXCR4^+^ GC cells are affected by the activation of TGF-β signaling. The RT-PCR experiment showed a decrease of *CXCR4* mRNA in 60As6 by TGF-β treatment (Fig 6Aii). Flow cytometry analyses reveal that TGF-β treatment diminish CXCR4^+^ small cells from 6.3% to 0.4% and to increase CXCR4^-^ large cells from 17.1% to 43.2% (Fig 6B). In accordance with an increase of terminally differentiated cells, caspase 3/7 in the whole cell population was activated by TGF-β treatment (Fig 6C). Therefore, TGF-β may have a potential to induce CXCR4^+^ CSCs to terminally differentiated cells. We further investigated the effect of TGF-β on quiescent 60As6 cells, since CSCs are thought to exist in a dormant state under a hypovascular condition. After serum depletion for 5 days, the CXCR4^+^ small cells increased up to 42% (Fig 6Di). In addition, a significant proportion of the 60As6 population was in G1 phase (Fig 6Dii). After the enrichment of CXCR4^+^ dormant GC cells by serum depletion for 2 days, we further treated them with 2 ng/ml TGF-β in a serum-free medium for additional 3 days. As a result, TGF-β diminished CXCR4^+^ small cells from 42% to 10% and increased CXCR4^-^ large cells from 8% to 26% (Fig 6Ei). Furthermore, a significant fraction of cells in G1 phase shifted to S phase by TGF-β (Fig 6Eii). These results suggest that TGF-β was capable of inducing the asymmetric cell division required for differentiation of CSCs even in a dormant state.

Several studies reported that cytotoxic anti-tumor drugs used in conventional chemotherapy eliminate rapidly dividing cells exclusively [35]. In other words, slow-cycling CSCs are hard to eradicate by these drugs [15]. Therefore, we finally investigated the reinforcement of TGF-β on anti-tumor drugs via induction of differentiation of CSCs. As expected, the combined use of docetaxel (Doc) and TGF-β more effectively diminished 60As6 cells than did Doc alone (Fig 6F). Same results were obtained in the primary culture of the ascites-derived GC cells from two independent patients (NSC-16C and -22C) (S7 Fig).

## Discussion

Cellular heterogeneity has been recognized as a defining feature of many leukemias and solid tumors. The CSC model postulates such a hierarchical organization of cells in which only a small subset establishes the cellular heterogeneity and is responsible for sustaining tumorigenesis. Since Dick and his colleagues first showed the presence of CSCs in AML [36, 37], the existence of CSCs in various types of solid tumors [38], which share some characteristics with adult tissue stem cells, such as cell differentiation ability, drug resistance and dormancy, has been demonstrated. Therefore, development of a new therapeutic strategy targeted to the dormant CSCs is eagerly awaited. However, because of cell plasticity and dynamic switching from non-CSCs to CSCs [17], determining whether any biological properties are relatively consistent in CSC populations, irrespective of any disease stage or individual tumor population, has been proven to be a challenge. Cell differentiation therapy has a long history of research and development, but the identification and understanding of CSC seem to cast a new therapeutic promise to overcome the hierarchical organization and the tumor cell plasticity.

In the present study, we initially focused on PD of diffuse-type GC cells and enriched the PD-associated CSCs by repetitive *in vivo* selection [24, 25]. Intriguingly, despite the fact that we did not use any anti-tumor drugs in the process of establishing a subline by the *in vivo* selection, our newly-established subline 60As6 possesses many of the properties of CSCs including a 6-fold higher drug resistance compared to its parental cells (S2 Fig). Moreover, lipid rafts accumulate in the cell membranes of this subline (S3 Fig). They have been reported to be enriched in hematopoietic stem cells [29] and to show a dynamic trafficking at invadopodia, which are necessary for invasive cancer cells to degrade the extracellular matrix in breast cancer [39].

By performing gene expression analyses, we showed that CXCR4 was upregulated in the highly tumorigenic subline compared to the parental one (Fig 1A and 1B). Interestingly, FACS analyses revealed a distinct heterogeneity in the CXCR4 expression level and in cell morphology within the population (Fig 1C). The CXCR4^+^ population was enriched for small cells, reportedly a typical morphological feature for melanoma cells with CSC phenotype [40]. Although CXCR4 overexpression has been reported in several human cancers [41–43], this is the first study to reveal CXCR4 is closely related to the CSC phenotypes of diffuse-type GC that has a high PD ability.

Consistent with previous reports [31], we showed that PD of diffuse-type GC cells was mediated functionally by CXCR4 activation, which may lead to the chemoattractive invasion of free cancer cells to SDF-1-expressing mesothelium (Fig 5). Furthermore, CXCR4^+^ small cells have several CSC-like properties including a repopulating ability (Fig 2A–2C) and high tumorigenicity (Fig 3B). Although shRNA-based knockdown of CXCR4 attenuated SDF-1-mediated migration but did not affect to the tumor nodule-formation ability (not shown), CXCR4^-^ population were decreased tumorigenesis and a colony formation ability (Fig 3A and 3B). These data indicates that the presence of unknown markers which are dictate other phenotypes of metastatic gastric CSCs (*e*.*g*. tumorigenesis) in CXCR4^+^ cell population. Thus, our studies provide novel insights into the roles of CXCR4 for PD and as a marker of metastatic CSCs.

Although TGF-β produced by cancer cells and/or by cancer-associated fibroblasts enhances the fibrosis, the role of TGF-β in the development of diffuse-type GC remains controversial. Either increased or decreased survival in diffuse-type GC patients was reported to correlate with the expression of TGF-β [44, 45]. Calon et al. reported that TGF-β signaling promotes colon cancer progression through the modification of stroma cells but not cancer cells, in which TGF-β signaling is inactivated [46]. Furthermore, it has been reported that TGF-β pathway have two opposite natures in respect to regulating CSC population. This pathway has been reported to activate CD44^+^ breast CSCs, whereas it also diminish the population of tumor initiating cells of gastric and pancreatic cancer [34, 47, 48]. Our present findings demonstrated that TGF-β signaling induces CXCR4^+^ quiescent CSCs to re-enter the cell cycle, leading to cell differentiation (Fig 6). Taken together, it is plausible that the controversy surrounding TGF-β treatment in solid tumors is the result of dual functions for TGF-β, which acts in a context-dependent manner to promote tumor progression in stroma cells and cell cycle re-entry in quiescent CSCs. We therefore propose that to achieve durable remission, the development of a novel combination therapy of cytotoxic agents and TGF-β should be considered based on the new knowledge and insights on the CSC biology and pathology. In such a regimen, TGF-β may need to be used transiently at GC onset, along with, or followed by, a standard of regimens that target rapidly dividing cells.

In previous reports, therapeutic interventions to exhaust and eradicate quiescent cancer stem/cancer-initiating cells were proposed to boost the anti-tumor drug effects for leukemia [49, 50]. Importantly, we have shown here that this concept could also yield an improved therapeutic strategy for solid tumors. In fact, we detected the expression of CXCR4 and/or CXCR7 in cancer cells in the ascites of GC patients (S5B and S5C Fig) and suggested the clinical relevance of these cells by demonstrating their CSC-like features such as a repopulating ability. Our findings also call for the assessment of these chemokine receptors in isolated cancer cells from the peritoneal cavity as a possible biomarker for patient stratification (S5A Fig).

## Supporting Information

S1 Fig Colony formation assay of HSC-60 and 60As6. Representative images of anchorage independent growth of HSC-60 and 60As6 cells in a soft-agar plate (left). Quantitative analysis of cell growth using CyQuant GR dye (right) (n = 3, mean + SE; ***p<0.001). Scale bars represent 100μm.
(TIF)

S2 Fig Drug resistance of HSC-60 and 60As6. Representative phase-contrast images of each cell with Doc treatment. Both HSC-60 and 60As6 cells were cultured for 4 days in the presence of 0–100 nM Doc and detected the number of the viable cells by MTT assay. IC_50_ against Doc in HSC-60 and 60As6 is 8.4 ± 0.4 and 52 ± 9.8 nM, respectively (n = 3, ***p<0.001). Scale bars represent 100μm.
(TIF)

S3 Fig Morphology and lipid raft formation of HSC-60 and 60As6. Representative phase-contrast images of HSC-60 and 60As6 cells (upper). Cellular localization of a fluorescent-labeled cholera toxin B subunit, which binds to lipid raft-enriched GM1 ganglioside (lower). Scale bars represent 50μm.
(TIF)

S4 Fig Time-lapse imaging of CXCR4^-^ large cell subpopulation. Representative images of the cells in the CXCR4^-^ large cell subpopulation identified as the fraction III in Fig 1*c*i. This subpopulation contains the large cells (black square) as well as doublet and triplet small cells (red circle). The cell division of each cell was followed on Day 3 (right column).
(TIF)

S5 Fig Presence of CXCR4^+^ Cancer Cells in Various Clinical Samples. (A) RT-PCR of *CXCR4* and *CXCL12* in peritoneal washings of 44 gastric cancer patients with free cancer cells (CY^+^: n = 34) or without them (CY^-^: n = 10). (B) Immunocytochemistry for pankeratin (red) and CXCR4 (green) of GC cells attached to the cobblestone-shaped peritoneum in a short-term culture of the patient’s ascites. (C) Immunocytochemistry for vimentin, pankeratin, CXCR4 and CXCR7 of GC cells in a long-term culture of the patient’s ascites. Immunopositive cells for CXCR4 and CXCR7 are indicated by black arrowheads.
(TIF)

S6 Fig Expression of *TGFBR1* and *TGFBR2* in GC Cells. RT-PCR of *TGFBR1* and *TGFBR2* in 60As6 and the primary cultures of malignant ascites of the patients with GC (NSC-16C and -22C).
(TIF)

S7 Fig Combination treatment of Doc and TGF-β in primary cultured GC cells. The viability of NSC-16C and -22C cells 3 days after Doc treatment with or without TGF-β (n = 3, mean + SE; *p<0.01).
(TIF)

S1 Table Genes highly expressed in 60As6 (upper) and in HSC-60 (lower). (DOCX)
