# Supplementary figures and images for: Identification and Characterization of CXCR4-Positive Gastric Cancer Stem Cells
Source: PLoS One. 2015 Jun 25;10(6):e0130808. doi: 10.1371/journal.pone.0130808 (PMC4481351; doi:10.1371/journal.pone.0130808)

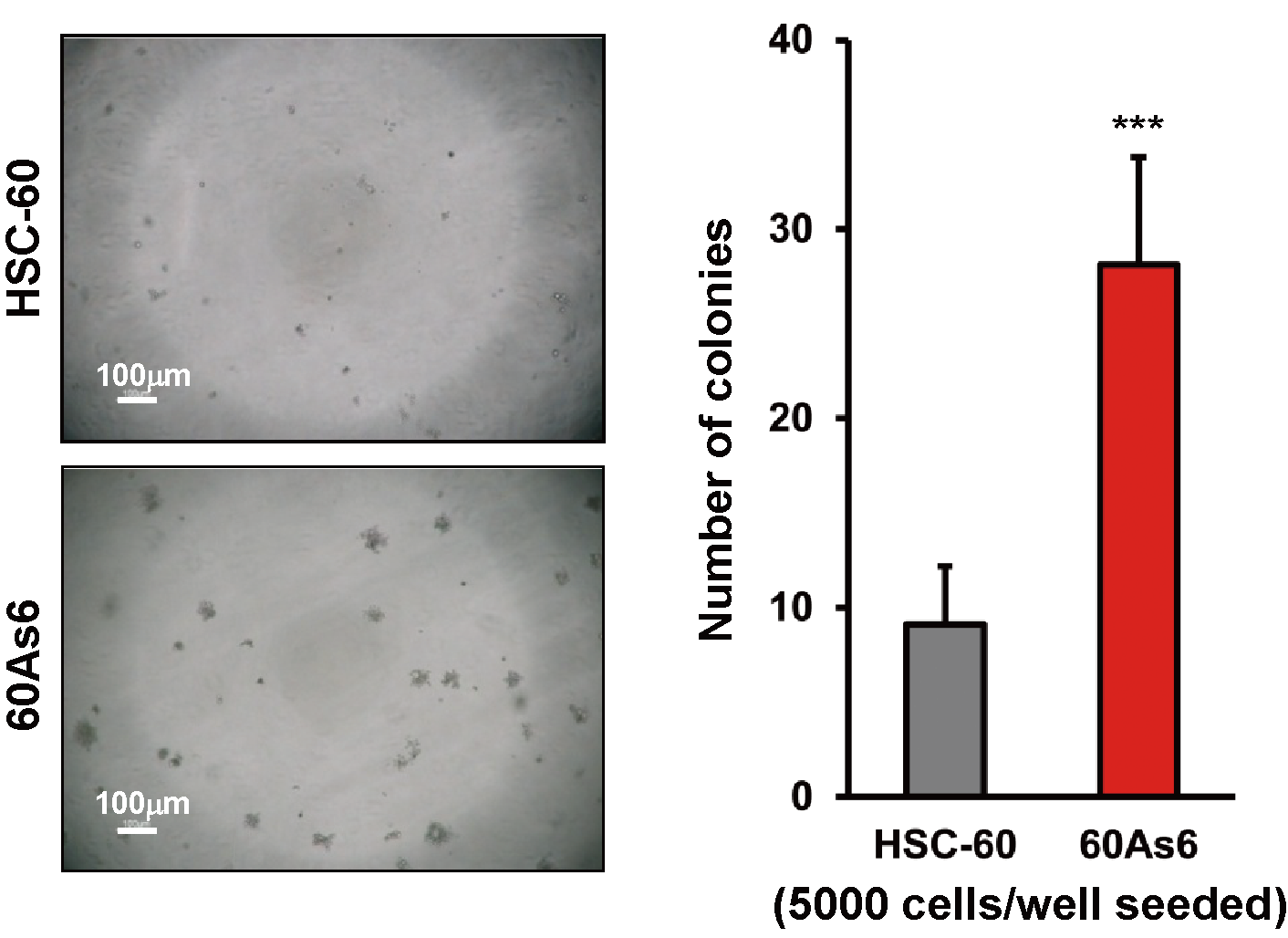

Supplement: S1 Fig — Representative images of anchorage independent growth of HSC-60 and 60As6 cells in a soft-agar plate (left). Quantitative analysis of cell growth using CyQuant GR dye (right) (n = 3, mean + SE; ***p<0.001). Scale bars represent 100μm. (TIF) [file pone.0130808.s001.tif]

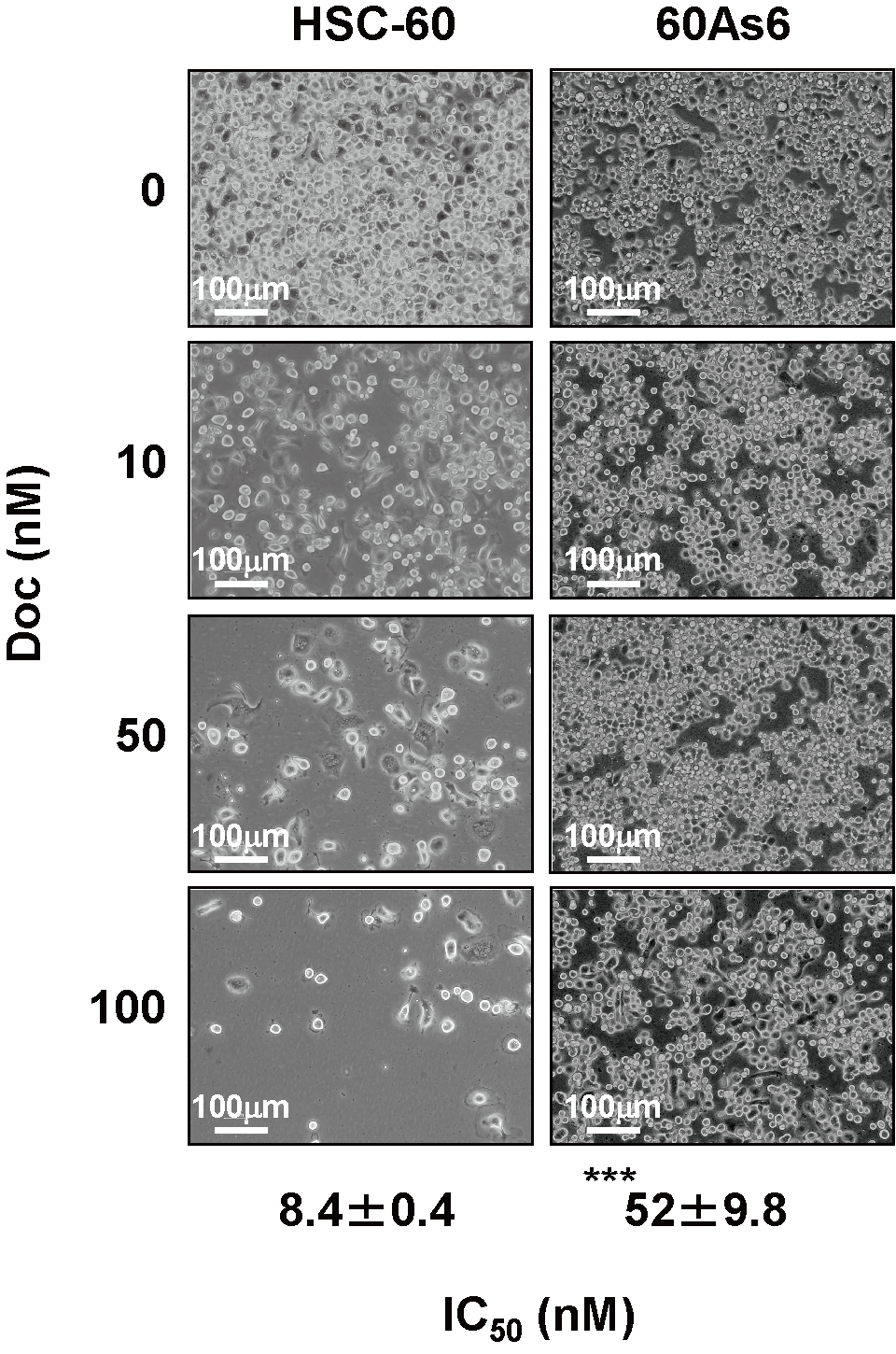

Supplement: S2 Fig — Representative phase-contrast images of each cell with Doc treatment. Both HSC-60 and 60As6 cells were cultured for 4 days in the presence of 0–100 nM Doc and detected the number of the viable cells by MTT assay. IC50 against Doc in HSC-60 and 60As6 is 8.4 ± 0.4 and 52 ± 9.8 nM, respectively (n = 3, ***p<0.001). Scale bars represent 100μm. (TIF) [file pone.0130808.s002.tif]

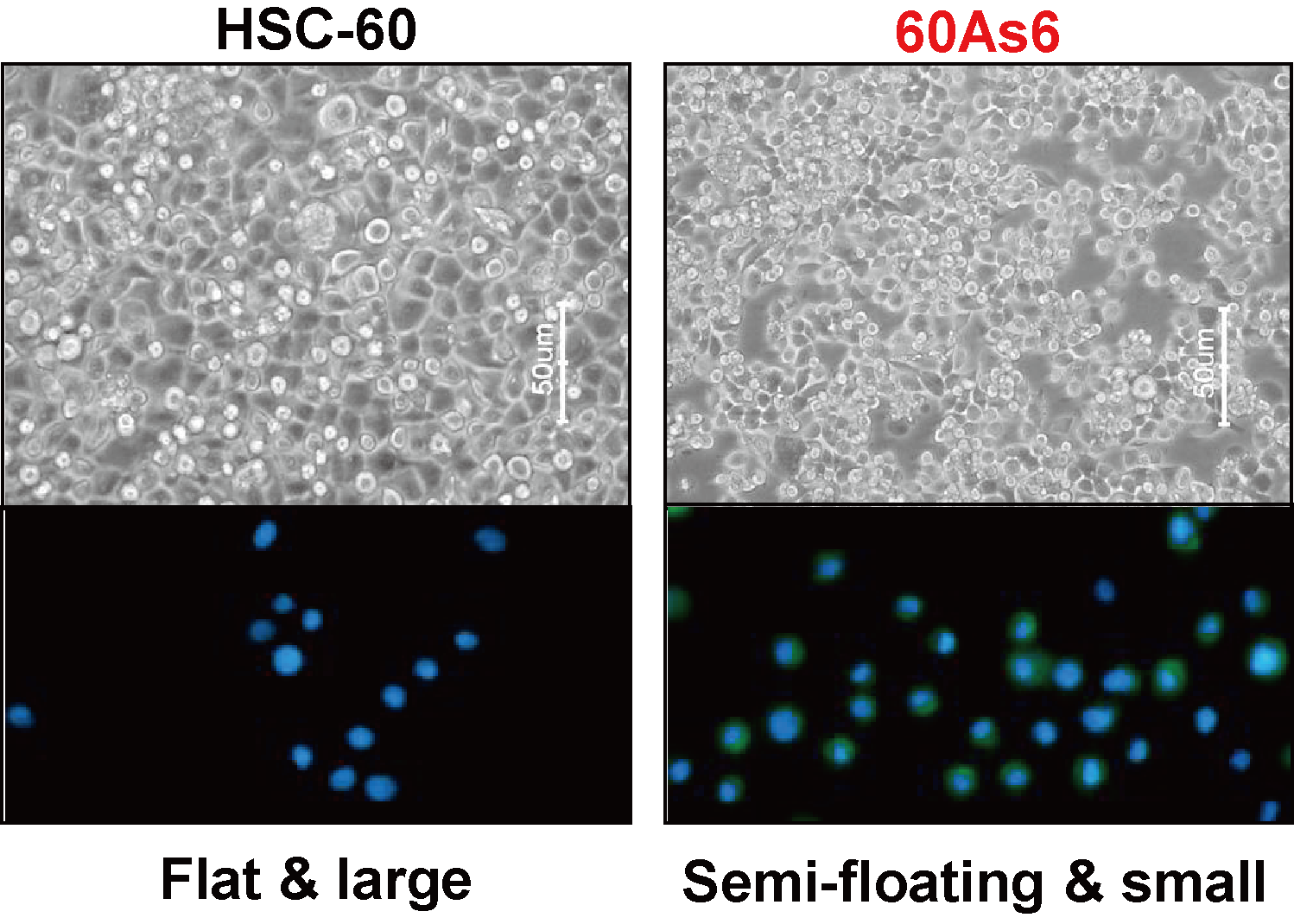

Supplement: S3 Fig — Representative phase-contrast images of HSC-60 and 60As6 cells (upper). Cellular localization of a fluorescent-labeled cholera toxin B subunit, which binds to lipid raft-enriched GM1 ganglioside (lower). Scale bars represent 50μm. (TIF) [file pone.0130808.s003.tif]

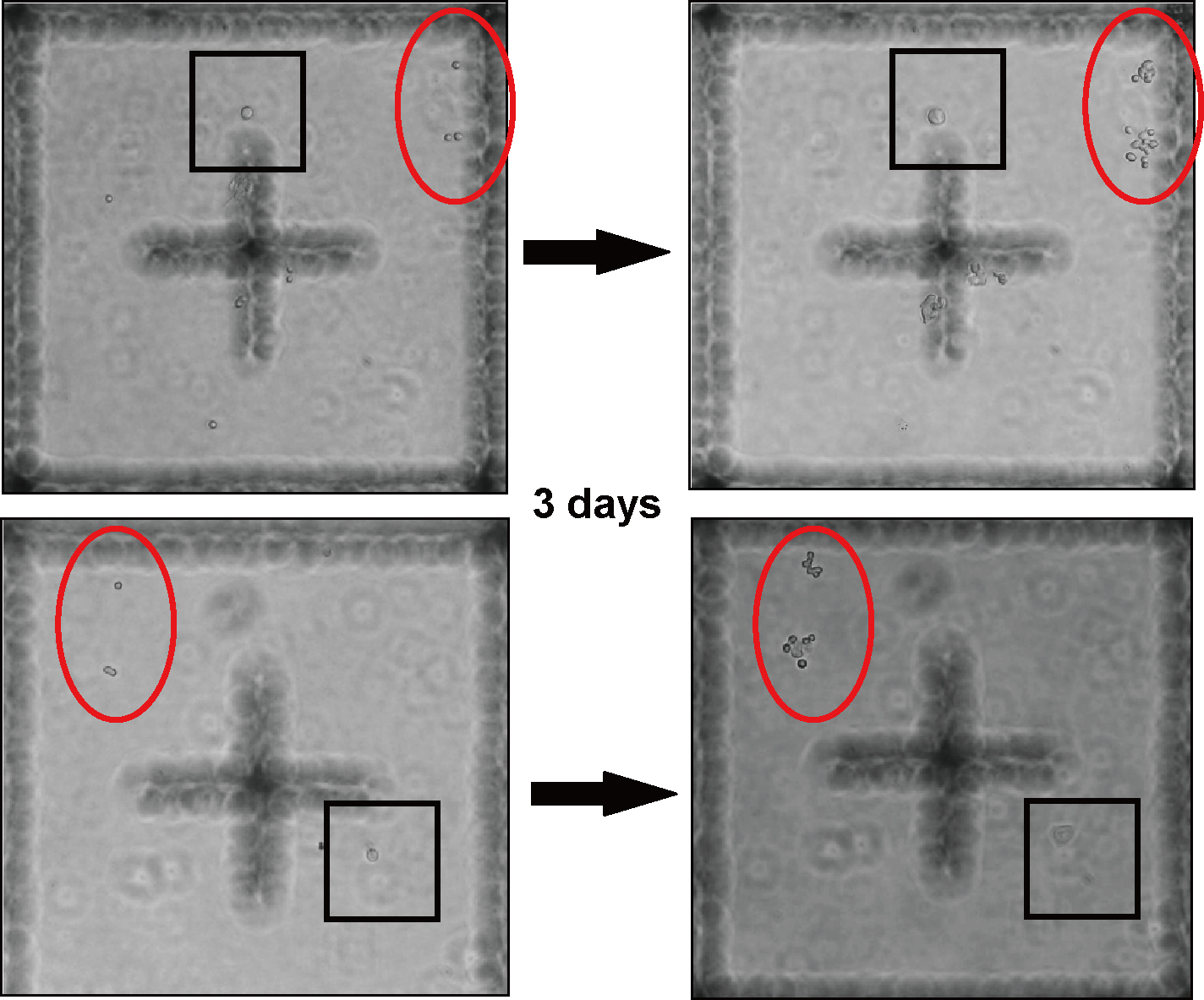

Supplement: S4 Fig — Representative images of the cells in the CXCR4- large cell subpopulation identified as the fraction III in Fig 1ci. This subpopulation contains the large cells (black square) as well as doublet and triplet small cells (red circle). The cell division of each cell was followed on Day 3 (right column). (TIF) [file pone.0130808.s004.tif]

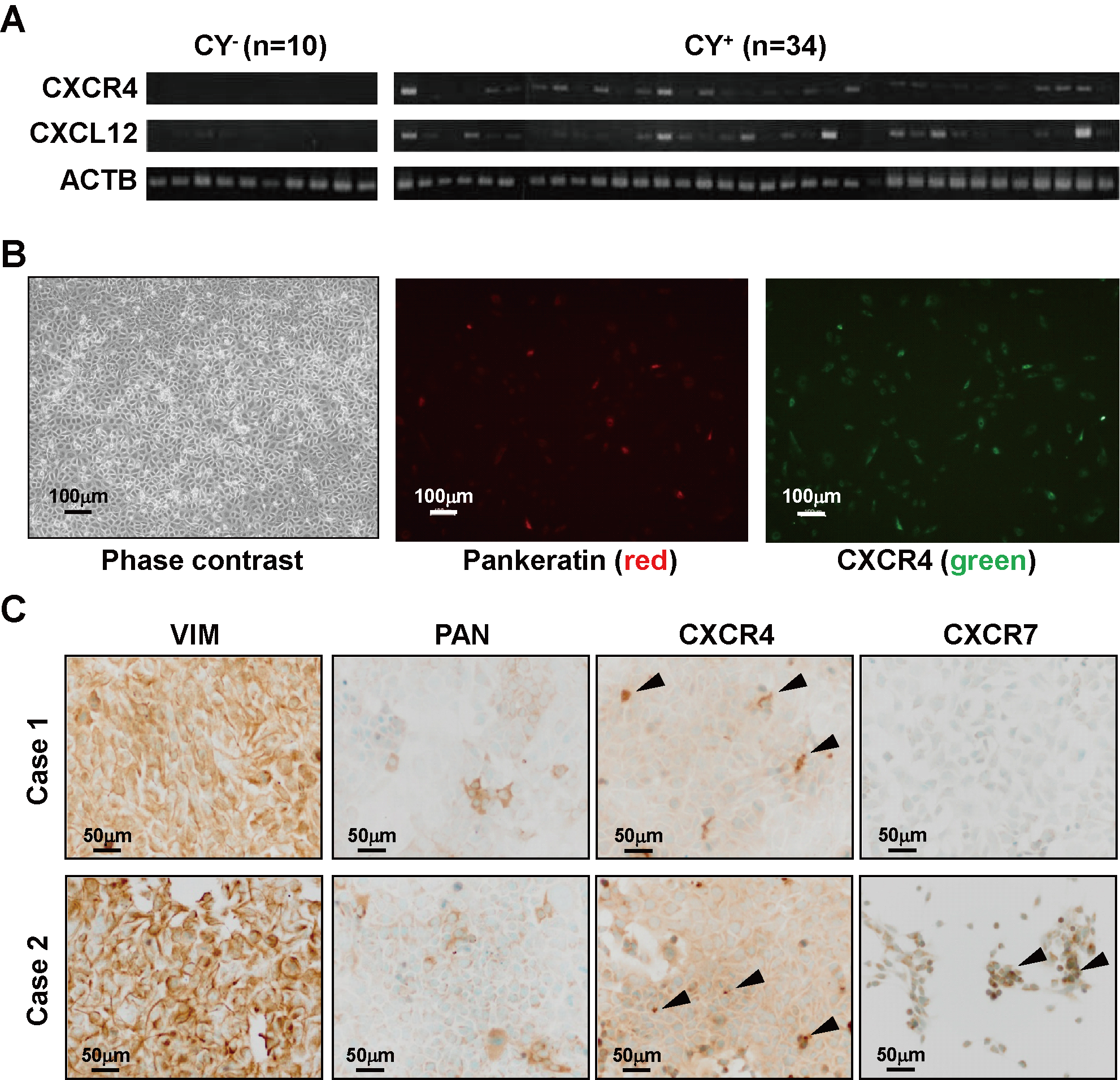

Supplement: S5 Fig — (A) RT-PCR of CXCR4 and CXCL12 in peritoneal washings of 44 gastric cancer patients with free cancer cells (CY+: n = 34) or without them (CY-: n = 10). (B) Immunocytochemistry for pankeratin (red) and CXCR4 (green) of GC cells attached to the cobblestone-shaped peritoneum in a short-term culture of the patient’s ascites. (C) Immunocytochemistry for vimentin, pankeratin, CXCR4 and CXCR7 of GC cells in a long-term culture of the patient’s ascites. Immunopositive cells for CXCR4 and CXCR7 are indicated by black arrowheads. (TIF) [file pone.0130808.s005.tif]

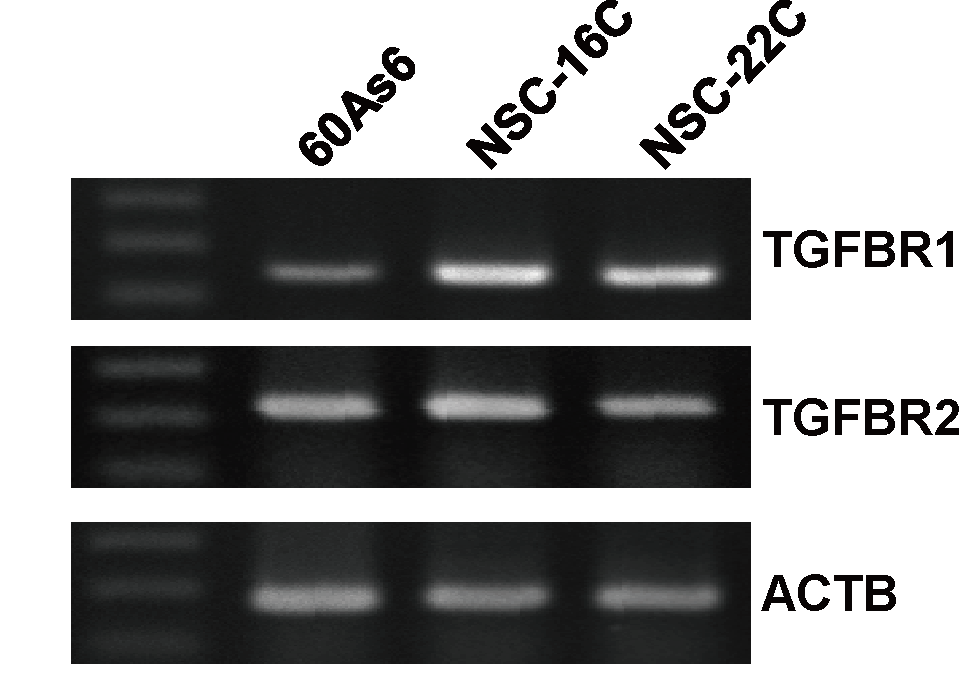

Supplement: S6 Fig — RT-PCR of TGFBR1 and TGFBR2 in 60As6 and the primary cultures of malignant ascites of the patients with GC (NSC-16C and -22C). (TIF) [file pone.0130808.s006.tif]

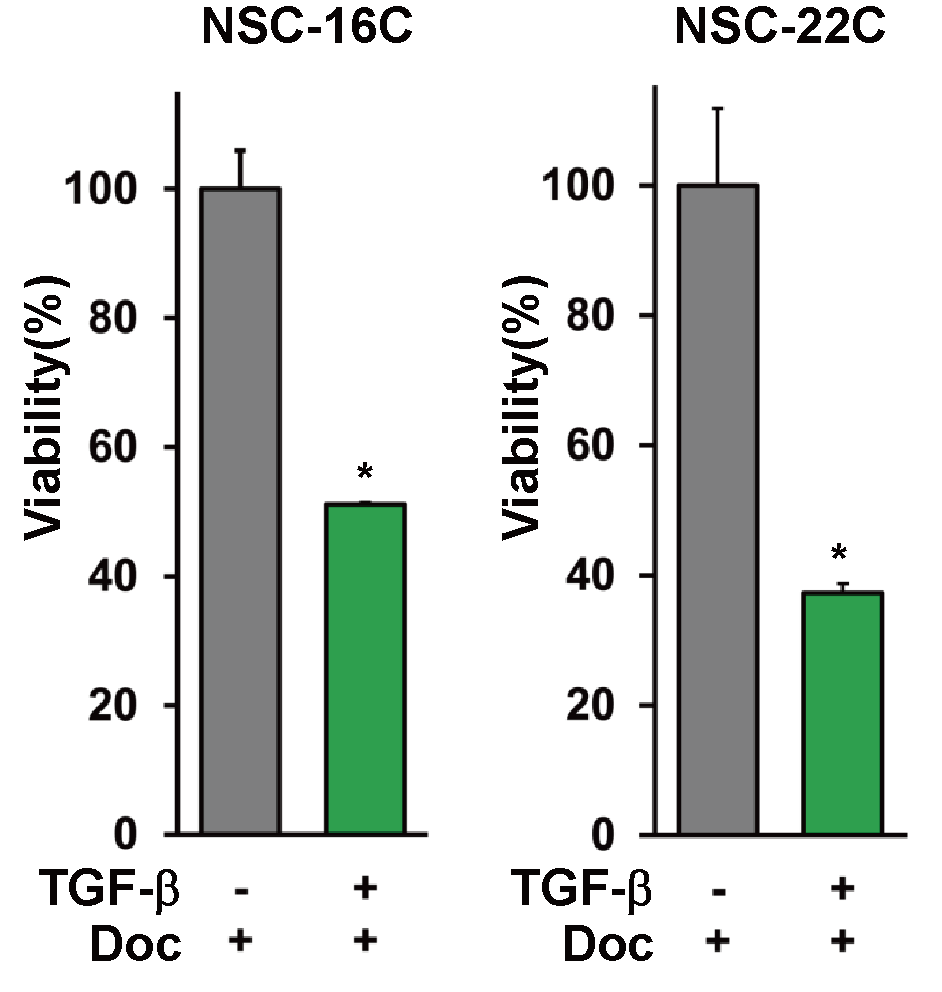

Supplement: S7 Fig — The viability of NSC-16C and -22C cells 3 days after Doc treatment with or without TGF-β (n = 3, mean + SE; *p<0.01). (TIF) [file pone.0130808.s007.tif]
