# Supplementary material for: Identification and Characterization of CXCR4-Positive Gastric Cancer Stem Cells
Source: PLoS One. 2015 Jun 25;10(6):e0130808. doi: 10.1371/journal.pone.0130808 (PMC4481351; doi:10.1371/journal.pone.0130808)
Supplement: S1 Table — (DOCX) [file pone.0130808.s008.docx]

**S1 Table. Genes highly expressed in 60As6 (upper) and in HSC-60 (lower)**

| Probe Set ID | Gene Symbol | Gene Title |
| --- | --- | --- |
| 205668_at | LY75 | Lymphocyte antigen 75 |
| 226545_at | CD109 | CD109 molecule |
| 217523_at | CD44 | CD44 molecule (Indian blood group) |
| 1554018_at | GPNMB | Glycoprotein (transmembrane) nmb |
| 217028_at | CXCR4 | Chemokine (C-X-C motif) receptor |

| Probe Set ID | Gene Symbol | Gene Title |
| --- | --- | --- |
| 204306_s_at | CD151 | CD151 molecule (Raph blood group) |
| 209772_s_at | CD24 | CD24 molecule |
| 201028_s_at | CD99 | CD99 molecule |
| 205157_s_at | KRT17 | Keratin 17 |
| 201596_x_at | KRT18 | Keratin 18 |
| 201650_at | KRT19 | Keratin 19 |
| 209008_x_at | KRT8 | Keratin 8 |
| 213693_s_at | MUC1 | Mucin 1, cell surface associated |
| 214303_x_at | MUC5AC | Mucin 5, cell surface associated |
| 205009_at | TFF1 | Trefoil factor 1 |
